# Coupling effects of deep vertical rotary tillage and brackish water irrigation on soil water-salt dynamics and cotton productivity in arid regions

**DOI:** 10.3389/fpls.2026.1787141

**Published:** 2026-03-02

**Authors:** Qiang Meng, Ning Su, Hongguang Liu, Pengfei Li, Tangang Wang, Rui Fang, Yongfu Wu

**Affiliations:** 1 College of Water Conservancy & Architectural Engineering, Shihezi University, Shihezi, China; 2 Key Laboratory of Modern Water–Saving Irrigation of Xinjiang Production & Construction Group, Shihezi, China; 3 Institute of Agricultural Science of the Third Division of Xinjiang Production and Construction Corps, Tumxuk, China; 4 Hydrology and Water Resources Management Center of the Second Division of Xinjiang Production and Construction Corps, Tiemenguan, Xinjiang, China

**Keywords:** cotton, deep vertical rotary tillage, multi-objective optimization, saline water drip irrigation, soil water-salt

## Abstract

**Introduction:**

Soil salinization poses an urgent challenge by constraining cotton yields and exacerbating freshwater scarcity.

**Methods:**

A two-year field experiment was carried out from 2023 to 2024. This study systematically assessed the effects of combining Deep Vertical Rotary Tillage (DVRT) with Saline Water Drip Irrigation (SWDI) on the soil salinity profile in the 0–100 cm layer, as well as on agronomic traits, fiber quality, yield, and water use efficiency (WUE) of cotton.

**Results:**

Results indicate that the DVRT-SWDI treatment significantly improved soil water storage capacity, among which the D3M2 treatment achieved the highest increment (12.06% higher than the control). Soil water storage exhibited a “decrease-increase-decrease” dynamic pattern throughout the growing season. Treatments with deep vertical rotary tillage (D2/ D3) effectively alleviated salt accumulation in the 0–60 cm root zone, with D3M1 showing the optimal salt-blocking effect, whereas shallow plowing combined with high-salinity water irrigation (D1M3) led to the most severe salt accumulation. Deep vertical rotary tillage integrated with low/medium-salinity water (M1/M2) significantly promoted cotton growth and yield formation. In 2023, the seed cotton yields under D2M2 and D3M2 treatments increased by 35.24% and 31.84%, respectively, compared with the control (CK). Comprehensive evaluation via the entropy weight method revealed that the D3M2 treatment delivered the highest overall benefit. Based on a multi-objective optimization model, the optimal combination of agronomic and tillage parameters was determined as a deep vertical rotary tillage depth of 40–45 cm and a saline water drip irrigation salinity of 2.82–3.29 g·L-^1^, which yielded seed cotton at 5767.38–6052.86 kg·ha-^1^.

**Discussion:**

This study clarifies the mechanisms of salt suppression and growth promotion by deep vertical rotary tillage under brackish water irrigation conditions, providing theoretical basis and technical support for the efficient utilization of water and soil resources and the suitable cultivation of cotton in saline-alkali lands of southern Xinjiang.

## 1 Introduction

Cotton (*Gossypium hirsutum* L.), as a key natural fiber raw material for the global textile industry, holds immense economic and strategic value. Approximately 25 million farmers worldwide are engaged in cotton production (Naoumkina and Kim, 2023; He et al., 2025). Xinjiang, as China’s largest cotton producing region, accounts for about 91% of the country’s total production (Kang et al., 2023). However, the high evapotranspiration and low precipitation climatic characteristics typical of the region, as well as the widespread saline soils, severely constrain cotton productivity and agro-ecosystem sustainability (Ge et al., 2022; Liu et al., 2025b). Statistics indicate that Xinjiang’s saline-alkali soil spans 21.844 million hectares, representing approximately one-third of the national total, with the Southern Xinjiang region bearing the brunt of this issue (Shao et al., 2025). In addition to this soil-related challenge, the region suffers from severe water scarcity: surface water availability is only 1/8 of the national average, and agricultural water use accounts for 95% or more of total water consumption (Li et al., 2020; Bian et al., 2024). Water scarcity and secondary soil salinization have become the two core bottlenecks hindering agricultural development in this region (Jia et al., 2025). Given that Xinjiang contains about 10 billion m^3^ of brackish water resources with mineralization greater than 3 g·L^-1^, and the mineralization of irrigation water in southern Xinjiang is generally between 2–6 g·L^-1^ (Bi et al., 2024). While the scientific use of brackish water for irrigation can alleviate freshwater crises and promote crop growth, it may also carry the risk of salt accumulation (Xiao et al., 2021; Wei et al., 2024). Therefore, exploring suitable micro-saline irrigation regimes is crucial for ensuring the sustainable development of agriculture in arid regions.

To address the dual challenges of water scarcity and soil salinization, advanced water-saving and salt-control technologies have been widely adopted in arid regions (Bian et al., 2024; Dong et al., 2025). Saline water drip irrigation (SWDI) effectively improves the water, thermal, and air environments in the crop root zone, playing a positive role in stabilizing and increasing crop yields (Xiao et al., 2021; Jiang et al., 2023). This technology employs a high-frequency irrigation pattern characterized by “small volumes at frequent intervals,” which enhances water use efficiency while temporarily suppressing salt accumulation in the surface soil layer of the root zone (Ma et al., 2024; Xu et al., 2024). However, under long-term high salt environmental stress, crop photosynthesis was inhibited, reducing water and fertilizer use efficiency and biomass accumulation (Ning et al., 2022; Feng et al., 2025; Zhao et al., 2026). At the same time, long-term application of SWDI may lead to inadequate soil salt leaching and increase the risk of salt accumulation in the deep root zone (Wang et al., 2019; Liu Y. et al., 2024), and further threaten the long-term stability of oasis agricultural systems (Guo et al., 2023; Wang et al., 2025).

How to effectively regulate soil salinity (SSC) in the root zone is the key to achieving safe use of brackish water (Duan et al., 2025; Guo et al., 2025). Although subsurface pipe drainage (SPD) technology offers advantages in lowering the water table, accelerating salt drainage, and improving soil pore structure (Chen et al., 2021; Li et al., 2024a; Wang et al., 2024), relying solely on SPD technology still faces bottlenecks in salt removal efficiency under brackish water irrigation conditions (Dou et al., 2022; Liu et al., 2025d). Deep Vertical Rotary Tillage (DVRT), as a novel tillage method, demonstrates remarkable effectiveness in breaking up the plow pan, improving soil physicochemical properties, promoting deep root penetration, and enhancing nutrient uptake (Li et al., 2024b). More importantly, DVRT can sever soil capillaries, block pathways for salt migration upward, and promote salt leaching into deeper soil layers (Yao et al., 2023; Li et al., 2025b).

Although existing research has demonstrated that SPD technology possesses a certain degree of salt rejection effect (Liu et al., 2025a). However, under high-salinity brackish water irrigation conditions, further research is needed to explore how to reduce root zone salinity risks. In particular, systematic studies are currently lacking on the combined effects and sustainability of the coupled system of Saline water drip irrigation (SWDI) and Deep vertical rotary tillage (DVRT) in mildly saline-affected cotton fields. Therefore, this study conducted a two-year (2023–2024) field-based positioning trial to investigate the integrated regulatory mechanisms of different SWDI and DVRT combination patterns. The primary research objectives include: (1) elucidating the distribution characteristics and accumulation patterns of soil water and salt under different technical combinations; (2) Reveal the impact mechanisms of this coupled model on cotton growth, development, and yield; (3) Conduct a multidimensional evaluation of different SWDI+DVRT combinations using the EWM-TOPSIS model to identify optimal saline water drip irrigation utilization patterns suitable for southern Xinjiang cotton fields. The findings will provide scientific basis and technical support for saline-alkali land remediation, safe utilization of saline water drip irrigation, and high-yield cotton cultivation in southern Xinjiang.

## 2 Materials and methods

### 2.1 Study site description

**Figure 1:**
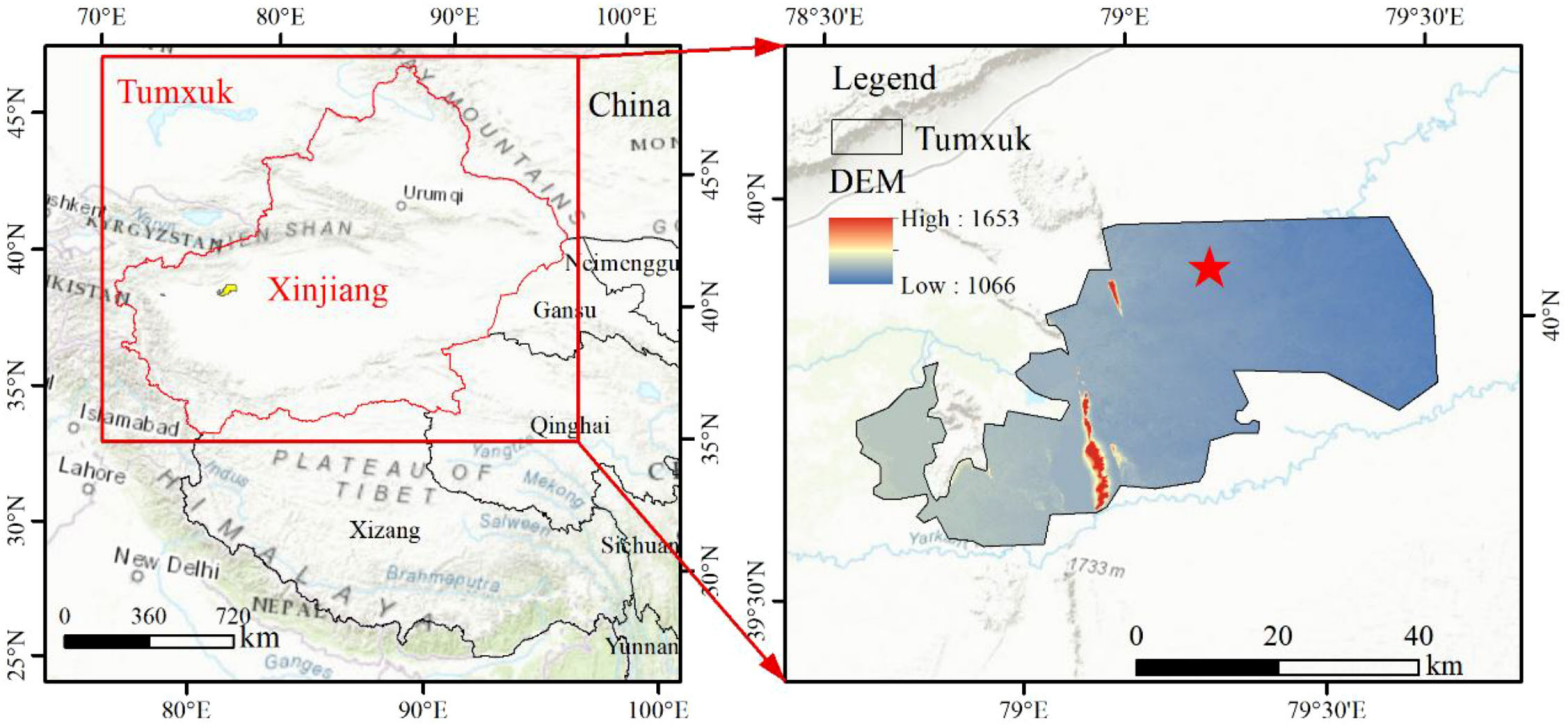
Schematic diagram of the location of the study area and the experimental arrangements.

**Figure 2:**
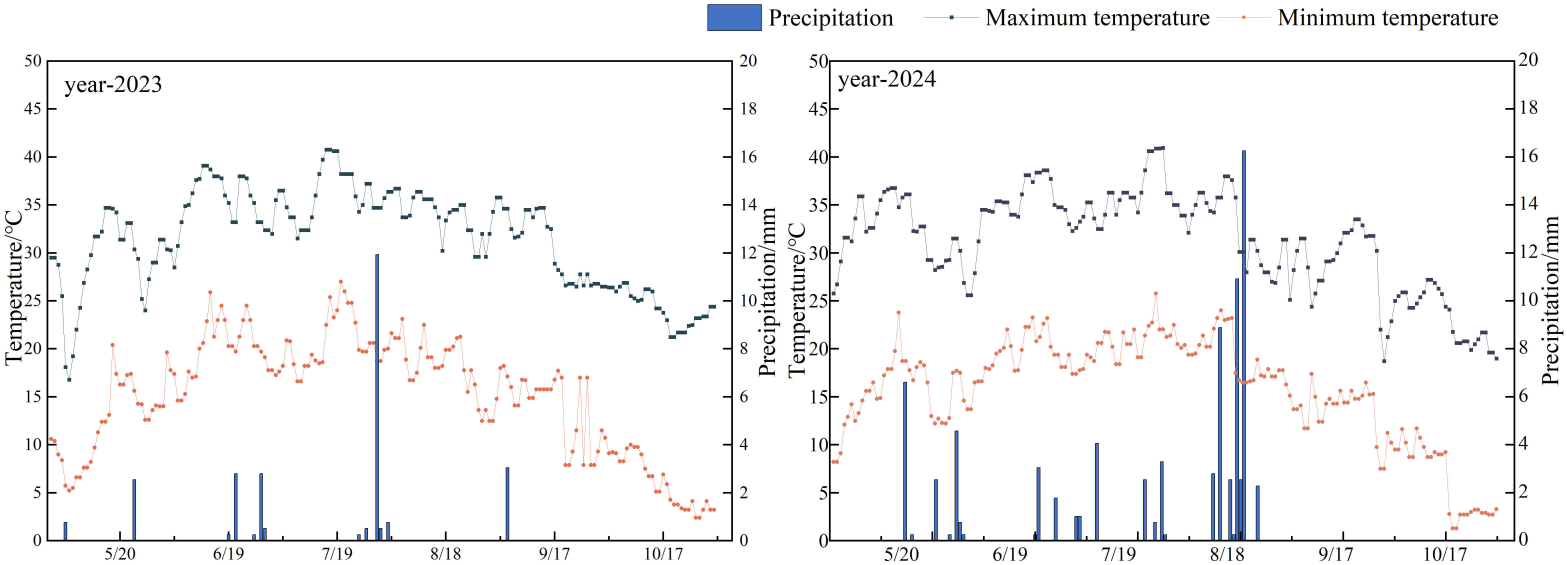
Precipitation and air temperature during cotton fertility in the study area.

This study was carried out in mildly salinized farmland in Tumushuke City, Xinjiang, China (79°2′8″E, 40°0′13″N, altitude 1098 m) (**Figure 1**). The study area is located at the western edge of the Taklamakan Desert, in the basin of the Yarkant and Kashgar Rivers, with a temperate extremely arid desert climate, with an average annual temperature of 11.6 °C, an annual precipitation of 38.3 mm, and an annual evapotranspiration of about 1643–2202 mm. The experimental period spans 2023–2024, from April to October each year, with an average rainfall of 32 mm and an average temperature of 23°C (**Figure 2**). Soil type is sandy loam, groundwater depth is 7 meters, and the bulk density of the 0–120 cm soil layer is 1.27 g·cm^-3^.The basic physical properties are shown in TableS1. In the 0–60 cm soil layer, the average content of soil organic matter (SOM) is approximately 12.98 g·kg^-1^,while the average contents of rapidly available phosphorus (APH), rapidly available potassium (APO), and alkaline nitrogen (ANH) are approximately 12.33, 240.65 and 36.86 mg·kg^-1^ respectively. For detailed information, please refer to **Supplementary Table S2**.

### 2.2 Experimental design

This study employed a two-factor completely randomized block design, incorporating three depths of deep vertical rotary tillage (DVRT) and three levels of saline water drip irrigationsaline water drip irrigation concentration (SWDI). Specifically, treatments included D1 (tillage depth 20 cm), D2 (tillage depth 40 cm), and D3 (tillage depth 60 cm), along with three mineralization levels of saline water drip irrigationsaline water drip irrigation:2 g·L^-1^(M1), 4 g·L^-1^(M2), and 6 g·L^-1^(M3),totaling six treatments. Each treatment was replicated three times, resulting in 18 plots arranged in a completely randomized block design. An additional CK treatment (conventional tillage with freshwater drip irrigation at 1 g·L^-1^)was included.

The buried pipes in this experimental area were installed at a depth of 1.0 m with 20 m spacing. Deep Vertical Rotary Tillage (DVRT) was conducted at varying depths in 2023 and 2024 using a deep vertical rotary tillage machine. Conventional tillage treatments employed a five-furrow plow from 2023 to 2024. Detailed experimental design is presented in **Table 1**. All treatments received spring irrigation (250 mm) post-tillage, with seeding conducted 7–10 days later. The experimental crop was cotton variety “Xinluzhong 75,” harvested annually in September.

**Table 1:** Experimental design: treatment combinations.

Treatment	Subsoiling depth (cm)	Brackish water salinity (g·L^-1^)	Treatment	Subsoiling depth (cm)	Brackish water salinity (g·L^-1^)
D1M1	20	2	D2M3	40	6
D1M2	20	4	D3M1	60	2
D1M3	20	6	D3M2	60	4
D2M1	40	2	D3M3	60	6
D2M2	40	4	CK	Conventional tillage 20 cm	1

**Figure 3:**
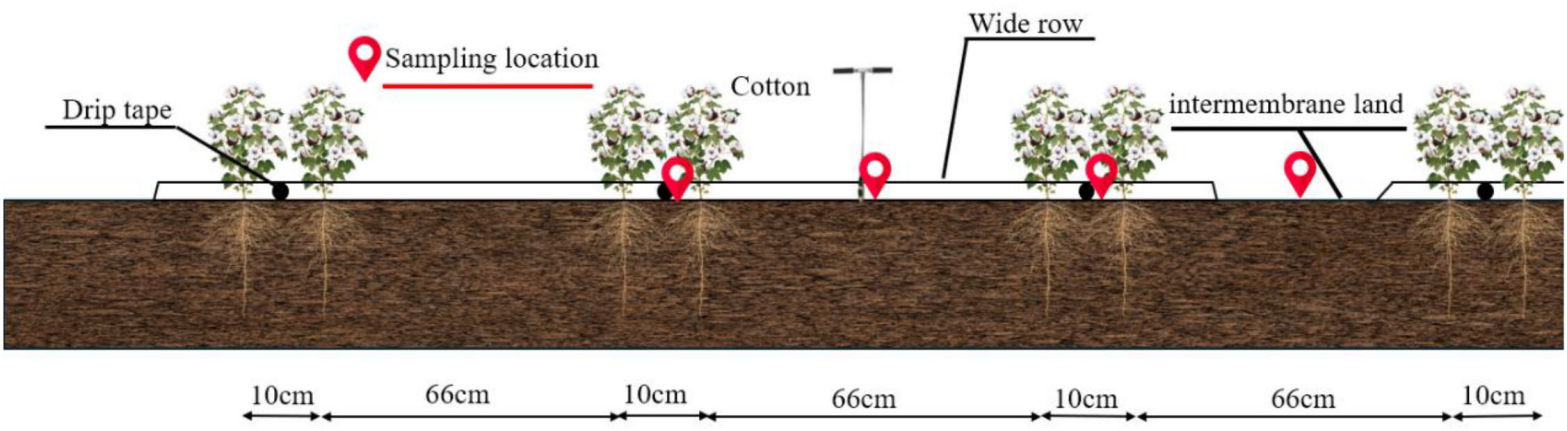
Cotton crop ping pattern in the test area.

Irrigation water sources comprised local deep groundwater (**Table 2**) and shallow brackish groundwater, with irrigation source details provided in **Supplementary Table S3**. A drip irrigation system under plastic mulch was employed, with drip tape arranged in a configuration of one mulch sheet per three pipes across six rows (**Figure 3**), laid perpendicular to the underground pipelines. Cotton was sown on April 26. Freshwater was used for seedling emergence irrigation. After entering the seedling stage on May 14, different water-salinity irrigation treatments were applied. A total of 10 irrigation events occurred during the cotton growth period. Irrigation regimes for the 2023 and 2024 cotton growing seasons are shown in **Supplementary Table S4**. Other agricultural management practices in the experimental area were identical to the high-yield management practices of surrounding farmlands. Throughout the entire experimental period in 2023 and 2024, all experimental methods and treatments remained consistent.

**Table 2:** Effect of DVRT and SWDI combinations on seedling emergence rate, crop quality, cotton dry mass, seed cotton yield.

Treatment	Seedling emergence rate (%)	Average length of above-ground section(cm)	Length Uniformity (%)	Specific strength at break (cN·tex^-1^)	Micronaire value	Elongation (%)	Cotton dry mass (kg·ha^-1^)	Seed cotton yield (kg·ha^-1^)
2023								
CK	76.17 ± 1.82e	27.0 ± 0.32a	83.5 ± 0.92a	25.4 ± 0.48a	4.9 ± 0.08ef	6.9 ± 0.18a	19080 ± 312def	4399 ± 125f
D1M1	78.36 ± 1.65de	26.5 ± 0.28abc	82.4 ± 0.85ab	24.0 ± 0.41c	4.8 ± 0.09f	6.8 ± 0.21a	19208 ± 298def	4725 ± 110de
D2M1	82.79 ± 2.53cd	26.9 ± 0.25ab	83.1 ± 0.78ab	24.8 ± 0.38ab	4.9 ± 0.07ef	6.9 ± 0.19a	19467 ± 285cd	4882 ± 135cd
D3M1	86.05 ± 2.72bc	27.0 ± 0.30a	83.3 ± 0.88ab	25.1 ± 0.45ab	4.9 ± 0.08ef	6.8 ± 0.17a	20418 ± 275b	5460 ± 142b
D1M2	83.85 ± 3.48c	26.0 ± 0.35cd	80.5 ± 0.95cd	22.8 ± 0.52d	5.3 ± 0.11bc	6.9 ± 0.20a	19893 ± 305bc	5146 ± 155c
D2M2	93.35 ± 2.61a	26.5 ± 0.31abc	82.1 ± 0.82abc	23.9 ± 0.46c	5.1 ± 0.10cde	6.8 ± 0.22a	21714 ± 332a	6248 ± 168a
D3M2	91.26 ± 2.57a	26.8 ± 0.29ab	82.8 ± 0.76ab	24.6 ± 0.40ab	5.0 ± 0.09def	6.9 ± 0.18a	21456 ± 318a	6091 ± 161a
D1M3	90.27 ± 1.70ab	25.5 ± 0.38d	79.8 ± 1.02d	21.6 ± 0.55e	5.7 ± 0.13a	6.6 ± 0.23a	18825 ± 290ef	4516 ± 138ef
D2M3	89.76 ± 2.55ab	26.3 ± 0.33bc	81.7 ± 0.89bc	23.2 ± 0.49cd	5.4 ± 0.12b	6.7 ± 0.20a	19440 ± 310cde	4867 ± 148cd
D3M3	89.66 ± 1.59ab	26.7 ± 0.27ab	82.5 ± 0.80ab	24.4 ± 0.42b	5.2 ± 0.11bcd	6.7 ± 0.19a	18687 ± 301f	4410 ± 130f
D	*	**	**	**	**	ns	**	**
M	ns	**	**	**	**	ns	**	*
D×M	ns	**	**	**	**	ns	**	*
2024								
CK	75.22 ± 2.29e	26.08 ± 0.41cd	82.50 ± 0.88bc	24.80 ± 0.66c	4.86 ± 0.15bcd	6.75 ± 0.21a	18810 ± 564f	5028 ± 135de
D1M1	76.50 ± 1.30e	26.80 ± 0.50bc	83.00 ± 0.86b	24.70 ± 0.75c	4.85 ± 0.15bcd	6.85 ± 0.21a	19949 ± 598def	5175 ± 155d
D2M1	78.00 ± 2.34de	27.50 ± 0.42b	85.50 ± 1.22a	25.80 ± 0.67bc	4.80 ± 0.14bcd	6.90 ± 0.21a	20232 ± 607cde	5347 ± 160cd
D3M1	79.50 ± 2.39cde	28.70 ± 0.34a	86.22 ± 1.08a	28.20 ± 0.79a	4.70 ± 0.14d	6.95 ± 0.21a	21274 ± 638bc	5980 ± 179b
D1M2	84.00 ± 3.22abc	26.12 ± 0.56cd	80.30 ± 1.25c	22.80 ± 0.54d	5.10 ± 0.15ac	6.85 ± 0.21a	20706 ± 621c	5635 ± 169c
D2M2	85.50 ± 2.27ab	27.56 ± 0.66b	82.10 ± 1.35bc	25.20 ± 0.76c	5.00 ± 0.15abc	6.90 ± 0.21a	22693 ± 681ad	6842 ± 205a
D3M2	87.00 ± 3.31a	29.39 ± 0.42a	83.20 ± 0.96b	27.60 ± 0.69a	4.85 ± 0.15bcd	6.90 ± 0.21a	22409 ± 672ab	6670 ± 200a
D1M3	81.80 ± 2.15bcd	25.22 ± 0.33d	81.20 ± 0.88bc	22.20 ± 0.73d	5.25 ± 0.16a	6.80 ± 0.20a	19002 ± 570ef	4600 ± 138f
D2M3	83.30 ± 1.20abc	26.31 ± 0.45c	82.50 ± 1.02bc	24.60 ± 0.94c	5.15 ± 0.15ab	6.80 ± 0.20a	19287 ± 579ef	4772 ± 143ef
D3M3	84.80 ± 2.24ab	27.70 ± 0.44b	82.80 ± 1.36b	27.00 ± 0.75ab	5.00 ± 0.15abc	6.85 ± 0.21a	19097 ± 573ef	4657 ± 140f
D	*	**	**	**	**	ns	**	**
M	ns	**	**	**	**	ns	**	*
D×M	ns	**	**	**	**	ns	**	*

D stands for depth of subsoiling, M stands for mineralization of brackish irrigation water, D×M represents the interaction between powdered ridge treatment and brackish water salinity; * and ** denote significance at the 0.05 and 0.01 levels, respectively; ns indicates non-significant. Error bars represent the standard deviation (n=3). Different letters indicate significant differences at *P* < 0.05.

### 2.3 Soil sampling and analysis

#### 2.3.1 Soil sample collection

Soil samples were collected vertically using an AMS Holland-type soil extraction auger 3 days after each irrigation when the soil moisture distribution was in a stable state. Soil samples were collected in the middle row of cotton (at the center line of the mulch), under the drip irrigation tape, and in the same cross-section of bare soil in the 0–100 cm soil layer, respectively, with the sampling location as in **Figure 3**, and were collected in 20 cm layers for split collection, and after mixing, soil electrical conductivity (EC, mS cm^−1^),and calculate the soil water storage capacity (SWS, g g^−1^).Sample collection was repeated three times at different locations for each treatment to ensure the reliability of the results.

Air-dry and grind the collected soil sample, then pass it through a 2 mm sieve. Weigh 20 g of the sample and use a conductivity meter (DDS-307, Shanghai Yinglian Scientific Instruments Co., Ltd., Shanghai, China) to determine the conductivity value of the soil extract prepared with a soil-to-water mass ratio of 1:5 (He et al., 2015). The pH was determined by potentiometric method with an acidimeter (pHS-2 type). The relationship equation between soil salinity and conductivity was calibrated by the dry residue method with the following Equation 1:

(1) $$\text{y}=4.5808\;{\text{EC}}_{1:5}-0.7886\;({\text{R}}^{2}=0.98)$$

where y is soil salinity,g·kg^-1^; ${\text{EC}}_{1:5}$ is the conductivity value, mS·cm^-1^.

Soil water storage is calculated using Equations 2, 3.

(2) $$SWC=\frac{{\text{m}}_{1}-{\text{m}}_{2}}{{\text{m}}_{2}}$$

(3) SWS=B×H×SWC×10

Where m_2_ is the weight of dried dry soil, g; m_1_ is the weight of fresh soil (g); B is the soil bulk density (g cm^−3^) was shown in **Supplementary Table S5**; H is the depth within the 0–100 cm soil profile (cm).

Soil salt accumulation is calculated according to **Equation 4**:

The soil salt accumulation rate (SSAR) represents the rate of increase in soil salinity within a given period relative to the preceding period. Its calculation formula is as follows:

(4) $$SSAR=\frac{W_{k}-W_{k-1}}{W_{k-1}}\times 100\%$$

Where SSAR is the soil salt accumulation rate,%;W_k_ is the soil salinity in period k, kg·ha^-1^;W_k-1_ is the soil salinity in period k-1, kg·ha^-1^.

#### 2.3.2 Measurement of cotton growth indicators

Samples were collected once during each growth stage: seedling stage, budding stage, boll formation stage, and lint shedding stage. For sampling, one uniformly growing cotton plant was selected (Wang et al., 2018). Plant height: Measure the distance from soil surface to cotton top using a tape measure, cm; Stem diameter: Measure the thinnest stem segment between the cotyledon node and the first true leaf using a vernier caliper, cm. Leaf area: Measure the longest and widest dimensions of the leaf using a tape measure (accuracy 1 mm). LAI was calculated by applying a standardized conversion factor (0.75) as established in previous studies, while the LAI was determined using the following **Equation 5**:

(5) $$LAI=0.75\rho \frac{\sum_{i=1}^{m}\;\sum_{j=1}^{n}\;L_{ij}B_{ij}}{m\times 10^{4}}$$

Where ρ represents the planting density (plants per square meter), m denotes the number of sampled plants, n represents the mean foliage count per individual specimen, and L and B indicate the maximum leaf length and width (in centimeters), respectively.

#### 2.3.3 Cotton seedling emergence and yield

The number of seedlings per plot was investigated from 20 d after sowing until flush, and one 3-m-long piece of discontinuous mulch was randomly selected for each replicated treatment in each experimental plot to calculate the seedling rate. The calculation formula is shown in Equation 6.

(6) $$\text{Seedling emergence}(\%)=(\frac{\text{number of seedlings}}{\text{number of seeds sown}})\times 100\%$$

Similarly, for each experimental plot and each replicate, select one 3-meter-long discontinuous plastic film strip. During the cotton boll-opening stage (September 28, 2023, and September 29, 2024), determine the number of effective plants, calculate the number of bolls per plant, weigh the effective bolls, and compute the average boll weight.

Seed cotton yield per unit area (kg·ha^-1^) = effective number of plants × number of bolls per plant × boll weight per boll.

Plant samples were collected from three 1 × 2 m sample plots randomly selected in each treatment area during the cotton harvest. The samples were dried at 105°C until constant weight was achieved, then dried further at 80°C for 3 days until completely dry. The dry weight was measured using a balance accurate to 0.01 g.

#### 2.3.4 Cotton fiber quality

After harvest, samples were selected in batches from each treatment and sent to the Ministry of Agriculture’s Cotton Quality Supervision, Inspection, and Testing Center for centralized determination of cotton fiber quality indicators using the HVICC (High-Volume Instrument Calibration Cotton) standard.

#### 2.3.5 Water use efficiency

Water Use Efficiency,(WUE,kg•m-3)is a core indicator for measuring the biomass or economic yield produced per unit of water consumed by crops, calculated using the Equations 7, 8:

(7) $$E_{T}=P+I+U-R-D-\Delta S$$

(8) $$WUE=Y/10E_{T}$$

Where WUE is the irrigation water use efficiency, kg·m^-3^;Y is the yield,kg·ha^-1^.P represents precipitation during the growing season, mm;I is the amount of irrigation water during the reproductive period, mm;U is groundwater recharge, mm;R is the surface runoff volume, mm;D represents the deep-layer leakage volume, mm;ΔS is the change in water storage in the soil profile at the beginning and end of the reproductive period, mm.

#### 2.3.6 Statistical analysis

In this study, one-way analysis of variance (ANOVA) was employed to analyze the effects of different treatment regimes on soil water storage capacity, soil salt content, crop plant height, stem diameter, leaf area index (LAI), crop quality, water use efficiency (WUE), and seed cotton yield. Duncan’s multiple range test (DMRT) was performed at a significance level of *P* < 0.05 to identify significant differences among the various treatments. Excel 2016 was used to collate the basic data of each indicator; Origin 2019 was applied to generate soil salinity interpolation maps and contour maps in the process of multi-objective optimization; the entropy weight method (EWM)-TOPSIS approach was utilized for multi-objective optimization; and MATLAB 2022a was employed for binary quadratic equation fitting and extreme value calculation.

## 3 Results

### 3.1 Soil water dynamics

**Figure 4:**
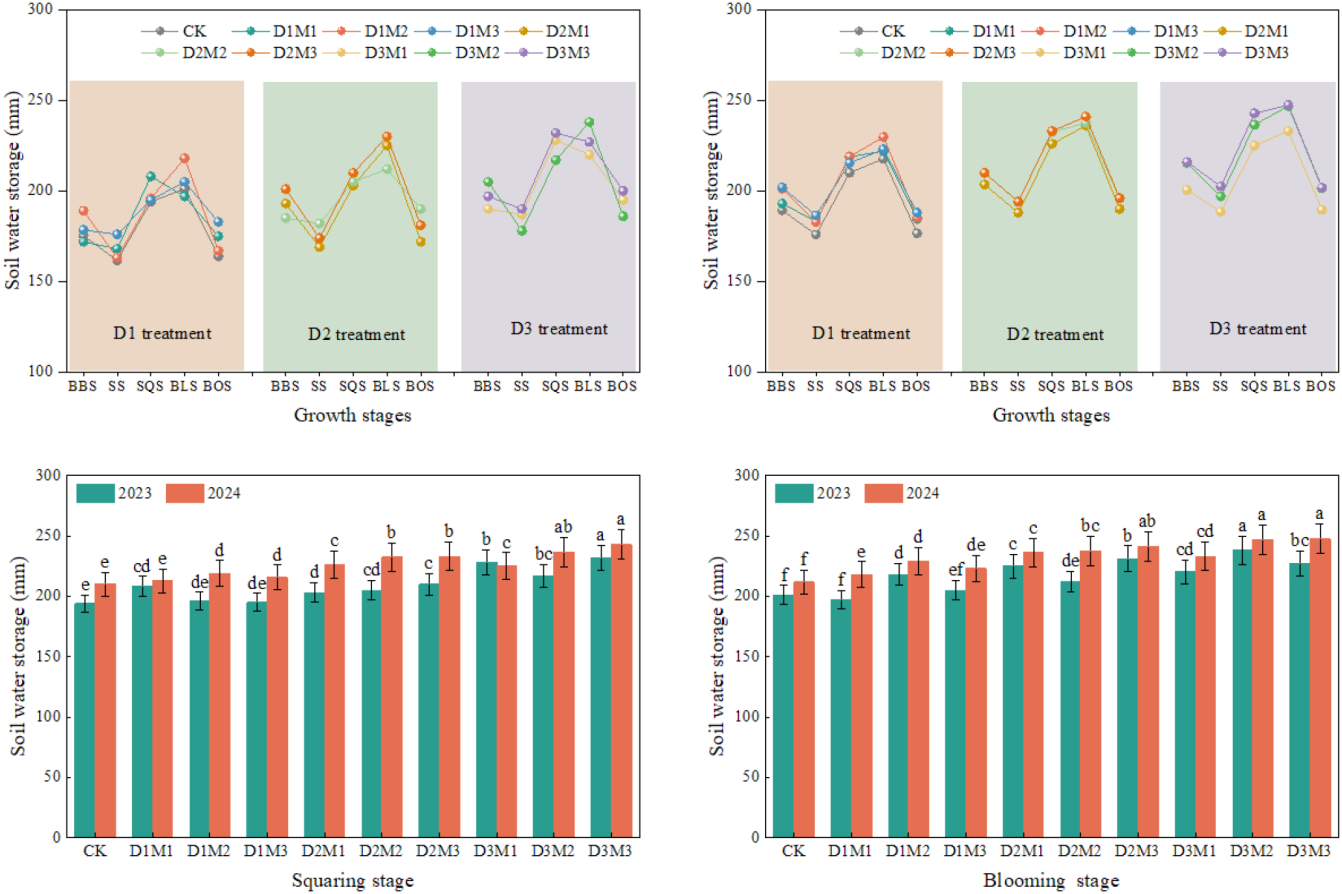
Effect of DVRT and SWDI combinations on soil water storage, 2023–2024. BSS (before seeding stage), SS (seeding stage), SQS (squaring stage), BLS (blooming stage), BOS (boll opening stage). Different letters indicate significant differences at *P* < 0.05. Lowercase letters indicate differences between different treatments at the same growth stage. Error bars represent the standard deviation (n=3).

**Figure 4** shows changes in soil water storage in the 0–100 cm soil layer during the cotton growing season from 2023 to 2024 for the combination of DVRT and SWDI. Results indicate that the combined application of DVRT and SWDI increases soil water storage, with D3M2 showing the highest increase of 12%. Among all growth stages, the soil water storage peaked during the BLS stage, and specifically, the storage under the D3M2 treatment reached 246.82 mm in 2024. Neither the CK nor D1 treatments exhibited statistically significant variations in soil water storage (*P* > 0.05). In contrast, relative to the CK treatment, the D2 and D3 treatments induced marked increases in soil water storage by 8% and 11% during the SQS and BLS stages, respectively.

Notably, spring irrigation sustained a relatively high level of soil water storage during the SS stage. However, a gradual depletion of soil water storage occurred during the BBS–SS stage, driven by active crop water uptake and the absence of irrigation events; during this period, the soil water storage under the D3M2 treatment was recorded at 197.36 mm. Conversely, during the SS–BLS stage, the cumulative irrigation amount (457.54 mm) exceeded the crop evapotranspiration (400.25 mm), which led to a progressive rise in soil water storage. Subsequently, during the BLS–BOS stage, soil water storage declined to 201.51 mm as a result of the peak water demand of cotton and continuous water consumption following the final irrigation event. Consequently, soil water storage exhibited a trend of initial decline, subsequent increase, and final decrease throughout the cotton growth period.

### 3.2 Soil salinity

**Figure 5:**
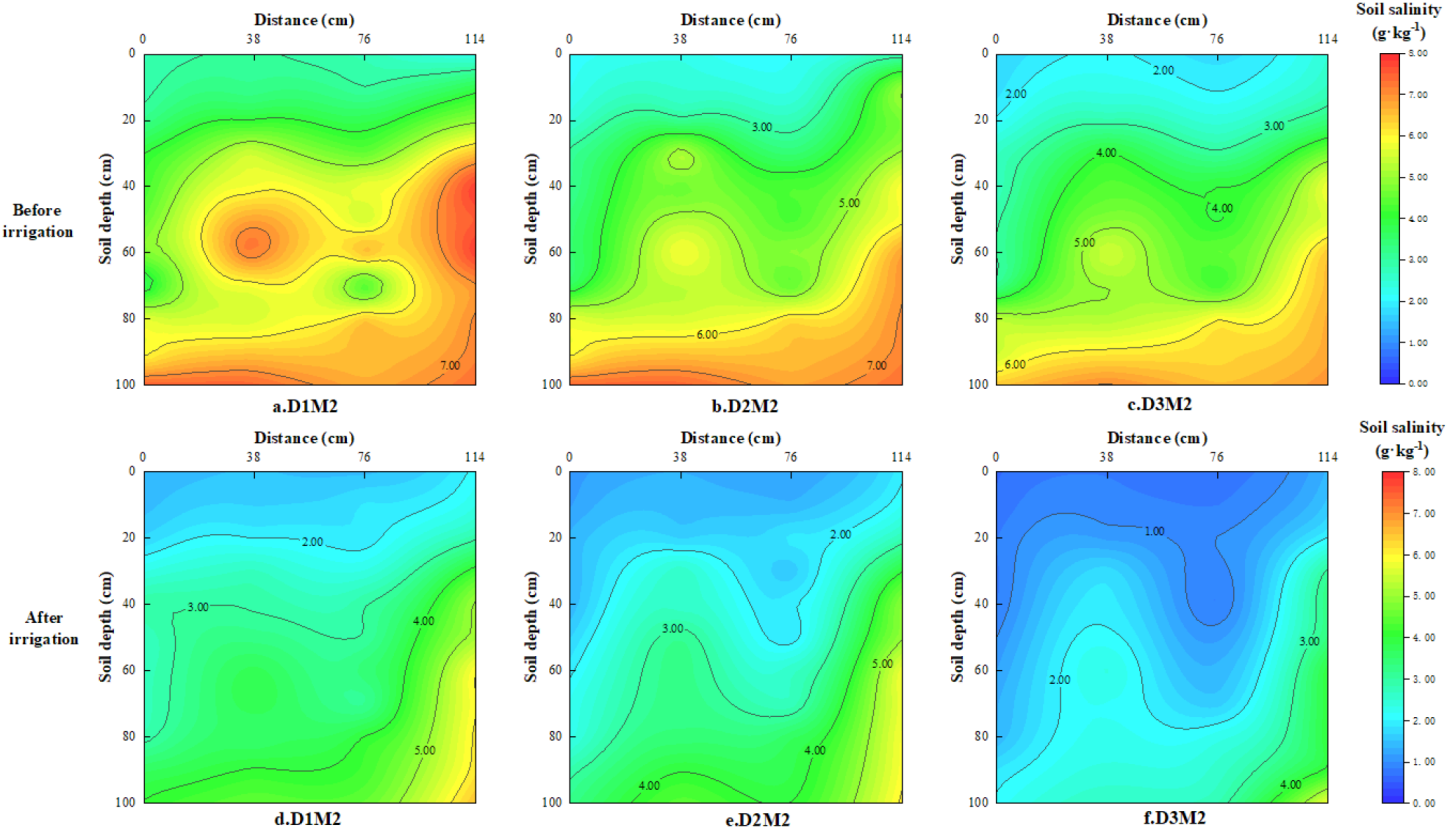
Soil salinity profile distribution at different powder ridge depths before and after irrigation under M2 treatment.

**Figure 5** demonstrates the profile distribution of salinity during irrigation at the cotton SQS stage. From **Figure 4a**, it can be seen that soil salts mainly accumulated in the deep soil layer 40–100 cm before irrigation in D1 treatment, and the average salinity of this layer was 2.8 times higher than that of the shallow layer 0–40 cm. In contrast, in **Figure 5b** and **Figure 5c**, the salt content in the 40–100cm soil layer of the D2 and D3 treatments was only 1.8 and 1.5 times that of the 0–40cm soil layer. From the horizontal dimension (**Figure 5a–c**), soil salinity was mainly distributed in the side rows and bare soil area, not in the crop root zone. As can be seen from **Figures 5d–f**, the average salinity in the 0–60 cm soil layer under the brackish drip irrigation treatment after flooding increased by 38.33%, 35.87%, and 23.72% in the D1, D2, and D3 treatments, respectively, compared with the CK treatment. As the operating depth of the deep vertical rotary tillage increased, the salt accumulation in the crop root zone (0–60cm) gradually decreased, and the desalination area expanded beyond the membrane.

**Figure 6:**
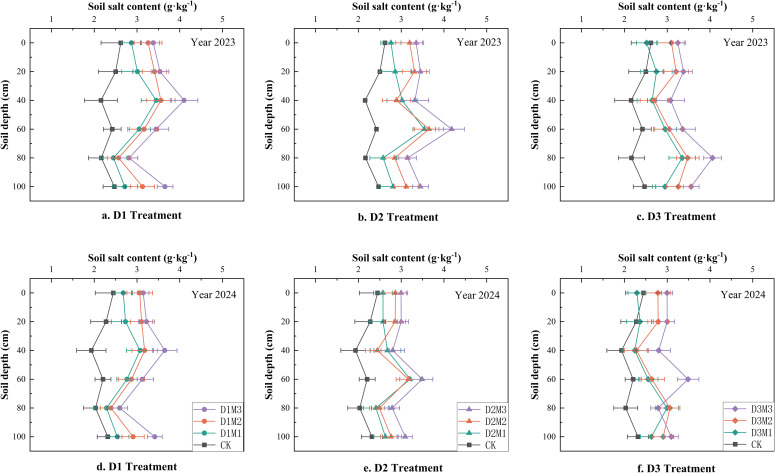
Distribution of soil salinity under different treatments, Error bars represent the standard deviation (n=3).

**Figure 6** presents the soil salinization rates under different treatments in 2023 and 2024. As shown in **Figure 6c** and **Figure 6e**, the D3M1 treatment exhibited the lowest salinization rates across the 100cm soil profile: 12.08% in the 0–60cm root zone and 19.39% across the entire 0–100cm profile. Conversely, **Figure 6a** and **Figure 6d** reveal that the D1M3 treatment exhibited the most severe salt accumulation across the 100 cm soil profile: the salt accumulation rate in the 0–60 cm root zone reached 49.16%, while the rate for the entire 0–100 cm profile was 45.02%. Due to excessive salt accumulation from the D1M3 treatment, the average salt content in the 0–100 cm soil layer reached 3.50 g·kg^-1^ and 3.61 g·kg^-1^(This result is consistent with the trends shown in **Figures 6a** and **6d**).

### 3.3 Crop growth indicators

**Figure 7:**
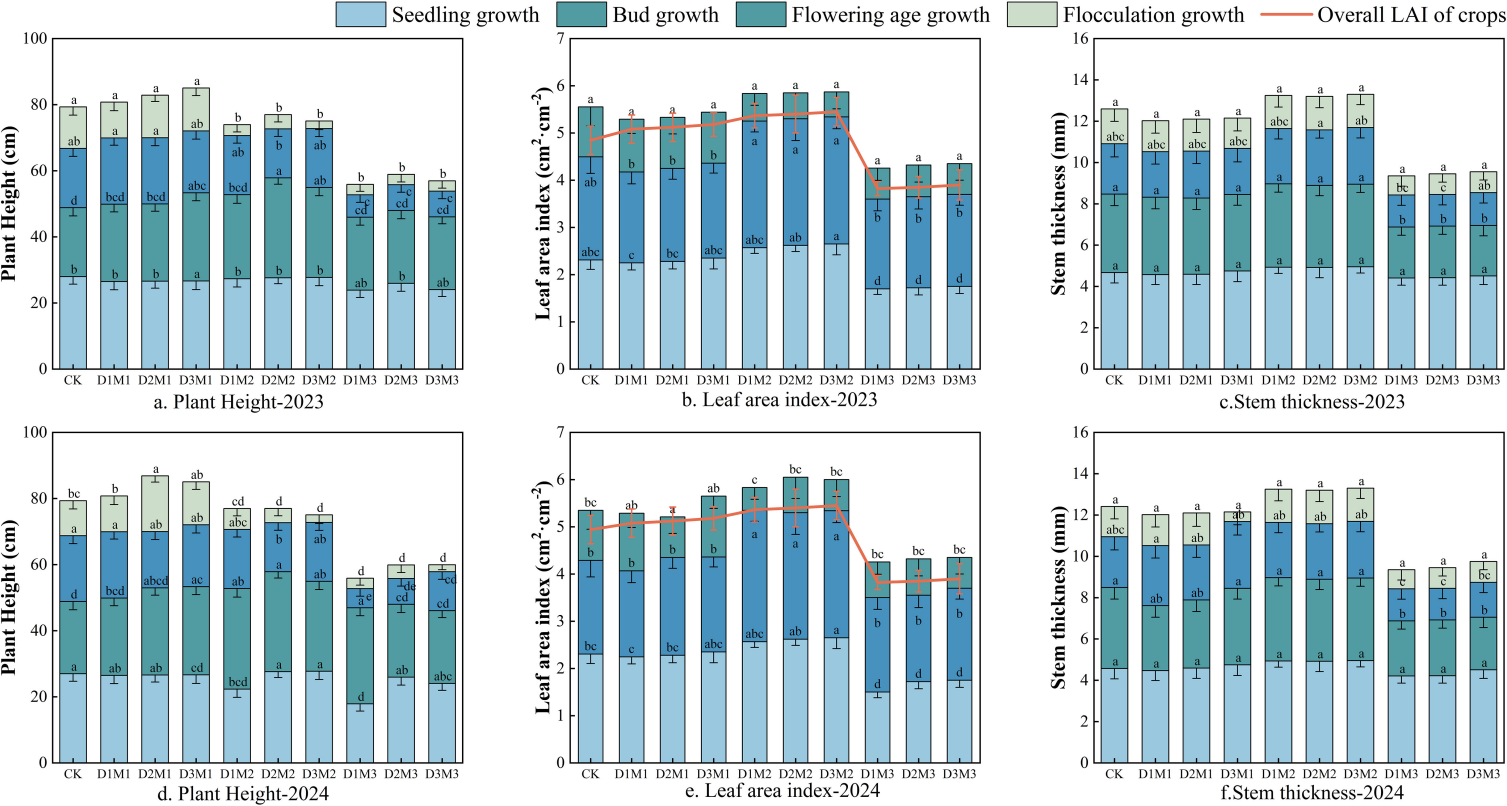
Effect of different treatments on height, stem thickness and leaf area index of cotton plants. Different letters indicate significant differences (*P* < 0.05). Lowercase letters indicate differences between treatments at the same growth stage. Error bars represent standard deviation (n=3).

**Figure 7** shows the growth dynamics of cotton plant height, leaf area index (LAI), and stem diameter under different Deep Vertical Rotary Tillage (DVRT) depths and Saline Water Drip Irrigation (SWDI) combinations in 2023 and 2024. Results for 2023 (**Figures 7a–c**) indicate that for plant height (**Figure 7a**), the D3M1 treatment exhibited the most pronounced promotion effect, with plant height throughout the growth period increasing by 5.71 cm compared to CK (*P* < 0.05). Conversely, the D1M3 treatment showed the strongest suppression, reducing plant height by 23.46 cm relative to CK. For LAI (**Figure 7b**), the D3M2 treatment exhibited the optimal growth rate, with a peak LAI of 5.45 during the boll-opening stage, 0.61 higher than CK (*P* < 0.05). Conversely, the D1M3 treatment showed a decrease of 1.02 compared to CK (*P* < 0.05). Regarding stem diameter (**Figure 7c**), the D3M2 treatment exhibited a stem diameter of 13.33 mm at the boll opening stage, which was 0.71 mm greater than CK (*P* < 0.05). Both the promoting effects of M1 and M2 treatments on stem diameter and the inhibiting effect of M3 treatment were statistically significant.

The treatment response trends for each indicator in 2024 were highly consistent with those in 2023 (**Figures 7d–f**). For plant height (**Figure 7d**), the D3M1 treatment remained the optimal combination, with the inhibitory effect of D1M3 continuing to be significant. The pattern of treatment differences in LAI (**Figure 7e**) and stem diameter (**Figure 7f**) fully replicated that of 2023. The combinations of M1, M2 with D2, D3 (medium/deep DVRT) consistently exhibited significant growth-promoting effects, whereas the combination of M3 with shallow DVRT (D1) exacerbated growth inhibition. Combining results from both years reveals a significant interaction between DVRT and brackish water: the combination of deep DVRT (D3) and low-mineralization brackish water (M1, M2) effectively mitigated the potential stress of brackish water, significantly improving cotton growth indicators. Conversely, high-mineralization brackish water (M3) paired with shallow DVRT (D1) produced a negative interaction, leading to a significant reduction in growth indicators.

### 3.4 Yield and fiber quality

In a two-year experiment, **Table 2** presents a comprehensive overview of key cotton growth and fiber quality parameters under the combined DVRT and SWDI treatments, including seedling emergence rate, average length of the above-ground section, Length Uniformity, specific strength at break, Micronaire value, elongation, cotton dry mass, and seed cotton yield. Compared with CK, in 2023, cotton seedling emergence was significantly higher (*P* < 0.05) under D2M2 and D3M2 treatments than under CK by 22.56% and 19.81%, respectively; mean above-ground length decreased by 1.85% and 0.74%, length uniformity by 1.68% and 0.84%, and ratio of breaks to strength by 5.91% and 3.15%, and macronaire value increased by 4.08% and 2.04%; in 2024, under the combination of DVRT and SWDI treatments, the changes in mean above-ground length, length uniformity, and specific strength of break over CK were -0.31%-2.76%, -0.61%-1.82%, -2.42% -1.61%, and the variation of Micronaire value was -3.29%-5.97%, which was significantly reduced by 3.29% and 1.23% under D3M1 and D2M1 treatments, respectively, compared with the control treatment (*P* < 0.05).

In terms of emergence rate, seed cotton yield of cotton plants, the combination of DVRT and SWDI treatments showed an increasing trend from year to year. However, in 2023, seed cotton yields under D1M3 and D3M3 treatments were significantly lower (*P* < 0.05) than CK by 2.25% and 4.55%, respectively; while seed cotton yields under D2M2 and D3M2 treatments were significantly higher (*P* < 0.05) than CK by 35.24% and 31.84%, respectively. Cotton dry matter accumulation under most of the DVRT and SWDI combination treatments was significantly higher than CK in 2023 and 2024, with D2M2 treatment increasing by 13.80% compared to CK in 2023, and this treatment increasing by 20.64% compared to CK in 2024. Seed cotton yield showed a similar trend, with the combination of DVRT and SWDI increasing by an average of 17.21% and 22.32% in 2023 and 2024, respectively, compared with CK; the combination of DVRT and SWDI treatments mostly showed a promotion effect on cotton growth and yield.

### 3.5 Soil salt accumulation rate and water use efficiency

**Table 3** presents the water use efficiency (WUE) and salt accumulation rate (SSAR) under different combinations of DVRT operating depth and brackish water irrigation treatments, and WUE showed a consistent response pattern in 2023 and 2024: the D2M2 treatment had the highest IWUE in both years (1.32a in 2023 and 1.43a in 2024), followed by the D3M2 treatment (1.27ab in 2023 and 1.39a in 2024). In contrast, WUE was significantly lower for the D1M3 and D3M3 treatments, suggesting that the combination of deep DVRT treatments (D2/D3) with low-mineralization brackish water (M1/M2) significantly increased WUE, whereas shallow DVRT treatments (D1) paired with high-mineralization brackish water (M3) resulted in a significant decrease in WUE.

**Table 3:** Response of water use efficiency and salt accumulation rate to deep vertical rotary tillage and saline water drip irrigation technologies.

Treatment	WUE (kg·m^-3^)		SSAR (%)	
	2023	2024	2023	2024
D1M1	0.98d	1.08d	0.28d	0.29d
D2M1	1.02cd	1.11cd	0.26de	0.27de
D3M1	1.14bc	1.25b	0.12g	0.11g
D1M2	1.07cd	1.17c	0.38c	0.37c
D2M2	1.32a	1.43a	0.35c	0.36c
D3M2	1.27ab	1.39a	0.24ef	0.25ef
D1M3	0.94d	0.96e	0.49a	0.50a
D2M3	1.10bc	0.99e	0.47b	0.48b
D3M3	0.92d	0.97e	0.35c	0.36c

Different lower-case letters in the graphs indicate significant differences between treatments at the *P* < 0.05 level.

Regarding salt accumulation rate, the D3M1 treatment exhibited the lowest salt accumulation over both years (0.12 g in 2023 and 0.11 g in 2024), while the D1M3 treatment recorded the highest SSAR values (0.49a in 2023 and 0.50a in 2024). This result further confirms the interactive effect between powder ridges and brackish water. Deep powder ridges (D3) combined with low-mineralization brackish water (M1) effectively mitigated soil salt accumulation, whereas shallow powder ridges (D1) paired with high-mineralization brackish water (M3) exacerbated salt accumulation in the soil profile.

### 3.6 Multi-Objective optimization based on spatial nalysis and TOPSIS

**Table 4** presents the TOPSIS comprehensive evaluation ranking based on four dimensions: crop growth, fiber quality, soil water use efficiency, and salt accumulation mitigation. The relative proximity (Hj) aligns with the interannual ranking results, indicating that the D3M2 treatment demonstrated the most optimal overall performance (ranked 1st in both 2023 and 2024), with values reaching 0.717 (2023) and 0.752 (2024), confirming that the combination of deep vertical rotary tillage and moderately mineralized saline water drip irrigation effectively balances multi-objective indicators. Conversely, the D1M3 treatment exhibited the poorest overall performance (ranked 9th in both years), aligning with the previously observed negative interaction effect between shallow deep vertical rotary tillage and highly mineralized brackish water. .

**Table 4:** TOPSIS comprehensive ranking based on crop growth, quality, soil water utilization and salt accumulation alleviation.

Treatment	2023				2024			
	D^+^	D^-^	H_j_	Rank	D^+^	D^-^	H_j_	Rank
D1M1	0.260	0.118	0.313	9	0.231	0.107	0.317	6
D2M1	0.226	0.159	0.412	5	0.194	0.162	0.455	4
D3M1	0.161	0.219	0.576	3	0.134	0.244	0.645	3
D1M2	0.199	0.151	0.432	4	0.187	0.150	0.444	5
D2M2	0.117	0.279	0.705	2	0.103	0.272	0.725	2
D3M2	0.109	0.275	0.717	1	0.089	0.269	0.752	1
D1M3	0.291	0.140	0.324	7	0.310	0.090	0.225	9
D2M3	0.229	0.133	0.368	6	0.284	0.089	0.238	8
D3M3	0.273	0.126	0.316	8	0.267	0.103	0.279	7

D^+^ denotes the euclidean distance to the positive ideal solution; D^-^denotes the euclidean distance to the negative ideal solution; H_j_ denotes the relative closeness to the ideal solution.

**Figure 8:**
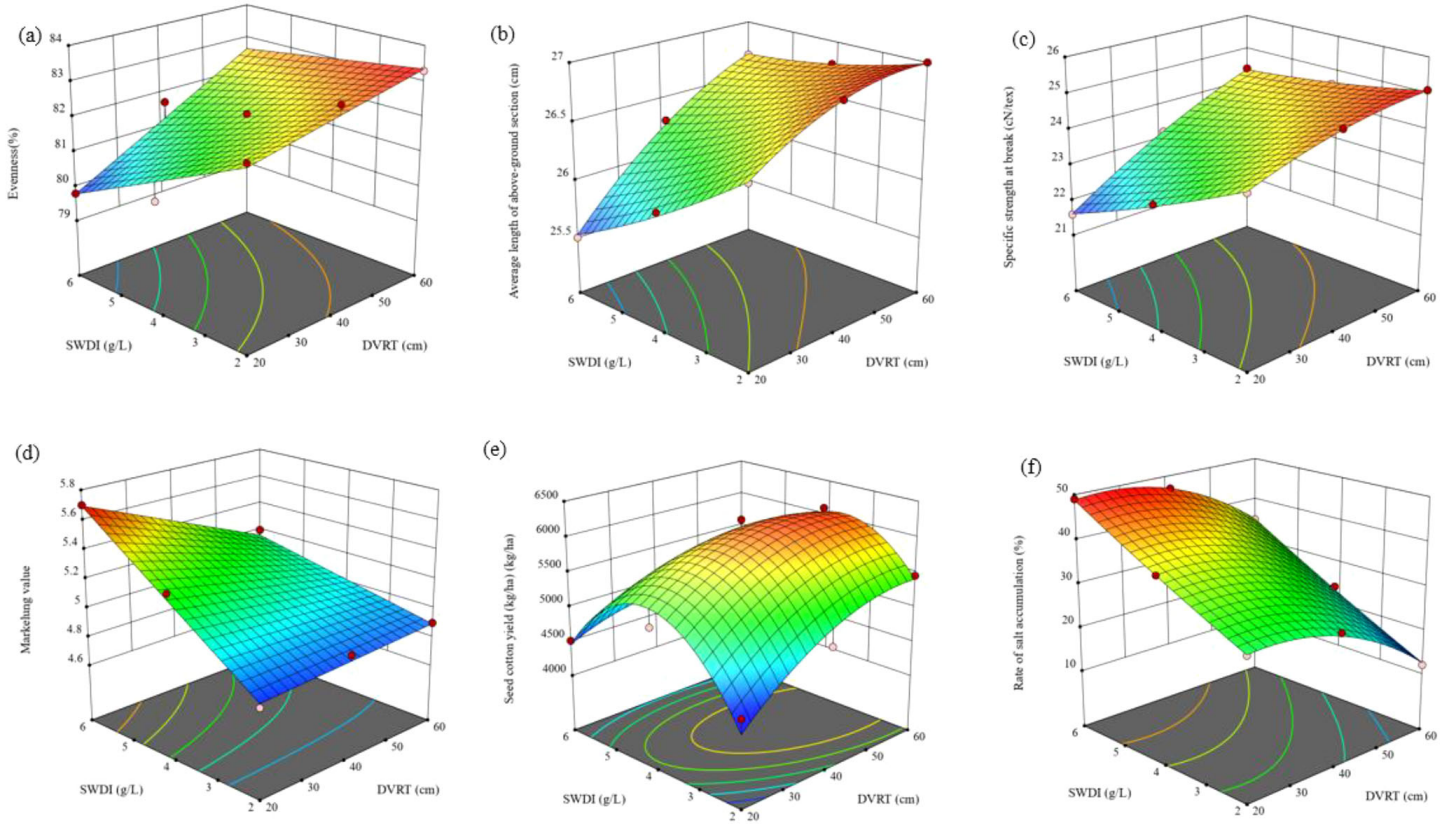
Relationships between various optimization indicators under different treatments.

To further quantify the optimal parameter ranges for deep vertical rotary tillage (DVRT) and saline water drip irrigation(SWDI) degree of salinity that balance the after mentioned indicators, this study employed response surface methodology (RSM) for multi-objective optimization (as shown in **Figure 8a–f**). The optimization results from **Table 5** indicate that the optimal parameter range is 40–45cm for deep vertical rotary tillage and 2.82–3.29 g·L-1 for saline water drip irrigation salinity index. Specifically, the optimal parameter combination achieved a cotton yield of 5767.38–6052.86 kg·ha-1, with a fiber micronaire value of 4.42. Additionally, the salt accumulation rate demonstrated effective soil salinity regulation, while quality indicators such as fiber uniformity, length, and specific strength exhibited balanced performance. These results confirm that this parameter combination simultaneously fulfills the multi-objective requirements of “high yield, high quality, and sustainable water-salt regulation”.

**Table 5:** Regression models of yield and t-values affected by Deep vertical rotary tillage (DVRT) and brackish water salinity.

Variant	Equation	*R* ^2^	*P*
Seedling emergence rate (Y1)	Y1 = 83.46 + 0.004D-0.850M+0.113DM	0.94	<0.05
Average length of above-ground section (Y2)	Y2 = 26.63 + 0.033D-0.433M+0.004DM-0.0004D^2^+0.0125M^2^	0.98	<0.05
Specific strength at break (Y3)	Y3 = 24.74 + 0.048D-0.983M+0.011DM-0.0005D^2^+0.0208M^2^	0.99	<0.05
Micronaire value (Y4)	Y4 = 4.21 + 0.009D+0.292M-0.004DM	0.98	<0.05
Seed cotton yield (Y5)	Y5=-1042.22 + 110.74D+2358.08M-7.29DM-0.822D^2^-268.17M^2^	0.94	<0.05
SSAR(Y6)	Y6 = 12.667 + 0.583D+3.917M+0.013DM-0.013D^2^+0.013M^2^	0.99	<0.05

D stands for depth of subsoiling, M stands for mineralization of brackish irrigation water, R² represents the regression equation coefficient of determination, and *P* represents the statistically significant value.

## 4 Discussion

### 4.1 Effects of deep vertical rotary tillage and saline water drip irrigation on soil water and salt transport

Deep Vertical Rotary Tillage (DVRT) is an innovative tillage practice that reconstructs soil architecture to ameliorate saline-alkali soils (Li et al., 2023, 2025a).Our finding indicate that the coupling of DVRT and SWDI significantly enhanced soil water storage in the 0–100 cm profile (Li et al., 2022). Specifically, during the critical reproductive stages (squaring and blooming), water storage capacity increased by 8%–11% compared to the CK treatment. This improvement is attributed to the ability of DVRT to shatter the compacted plow pan, thereby reducing soil bulk density and increasing total porosity. These structural changes enhance the infiltration rate of irrigation water and improve the soil’s water-holding capacity (Li et al., 2024b; Lin et al., 2025).

In terms of salt regulation, DVRT demonstrated a significant salt-blocking effect. As the tillage depth increased, the salt accumulation rate in the primary root zone (0–60 cm) declined, with salts migrating towards the deeper layers (40–80 cm). This aligns with Liu H. G. et al. (2024), who reported that deep tillage combined with leaching increased the desalination rate by 24.82%-44.17%. The mechanism lies in the high-speed rotary milling of DVRT, which transversely severs soil capillaries, effectively disrupting the upward migration of salts driven by evaporation (Lei et al., 2025). Furthermore, the “bulb-shaped” wetting front typical of drip irrigation facilitates the leaching of salts to the periphery of the root zone (Dong et al., 2018).

However, the mineralization of irrigation water remains a critical threshold. High-salinity water (6 g·L^-1^) led to salt enrichment in the 40–80 cm layer due to the limited leaching fraction of drip irrigation (Yang et al., 2021). In this study, the synergistic application of the sub-surface drainage (SPD) system mitigated risk. DVRT enhanced macropore flow, facilitating the vertical leaching of dissolved salts into the SPD system (Sharma et al., 2000; Liu et al., 2021). This “DVRT–Leaching–Drainage” nexus ensures periodic soil desalination, providing a sustainable soil environment for crop growth.

### 4.2 Effects of the interaction between deep vertical rotary tillage and saline water drip irrigation on cotton growth, yield, and fiber quality

The interaction between tillage practices and water quality significantly influences cotton productivity. Our results demonstrated that DVRT mitigated the inhibitory effects of salt stress, with treatments D2M2 and D3M2 increasing seedling emergence by 22.56% and 19.81%, respectively, relative to CK. This yield promotion can be explained by the physiological “compensation effect” resulting from an optimized root zone environment.

Mechanistically, the yield increase is likely driven by improvements in root morphology and nutrient uptake efficiency, although these specific indicators were not directly quantified in this study. DVRT significantly reduces soil mechanical impedance. Literature suggests that breaking the plow pan promotes the vertical proliferation of taproots and increases root length density in deeper soil layers (Rietz and Haynes, 2003; Huang et al., 2021). A deeper root system accesses subsoil water reserves, enhancing crop resilience during high-evapotranspiration periods. The desalination effect of DVRT alleviates osmotic stress. Lower soil salinity improves the water potential gradient between the soil and roots, facilitating water uptake and maintaining turgor pressure required for cell expansion (Zhang et al., 2020). This is corroborated by our observations of increased plant height and LAI under deep tillage treatments. Improved soil water storage enhances the mass flow and diffusion of nutrients to the root surface. Previous studies have indicated that DVRT enhances nitrogen accumulation in cotton biomass by optimizing the soil water-air ratio (Dong et al., 2025). Conversely, shallow tillage with high-salinity water (D1M3) exacerbated root zone salinization, likely inducing ion toxicity and hormonal imbalances which inhibited biomass synthesis (Maryum et al., 2022).

Water productivity and cotton fiber quality exhibit a pronounced trade-off relationship in response to water-salt stress. The interaction between deep vertical rotary tillage and Saline water drip irrigation directly influences water productivity by regulating soil water-salt content and structure (Zhang et al., 2024). Deep vertical rotary tillage enhances water infiltration capacity by reducing soil bulk density and increasing porosity; low-mineralization brackish water reduces salt input. Their synergistic action promotes salt leaching into deeper layers, alleviating root zone salinization. This improves root water uptake efficiency and growth vigor to boost yields, while their combined effect effectively increases water productivity. Conversely, shallow vertical rotary tillage and high-mineralization brackish water exacerbate salt retention in the root zone profile (Zhang et al., 2020). Increased soil osmotic pressure inhibits root water uptake, leading to reduced yields and increased ineffective water consumption, thereby decreasing water use efficiency. As the salinity of saline water drip irrigation increases, fiber quality indicators show trends of shorter length, decreased strength, and abnormal maturity (Ren et al., 2021). Specifically, the average length and uniformity index of the upper fiber portion decreased significantly, with breaking tenacity declining by 3.15%–5.91% and micronaire increasing by 2.04%–4.08%. From a physiological mechanism perspective, high salt stress compels cotton plants to prioritize carbon skeletons for synthesizing osmotic regulators (such as proline) to sustain survival, resulting in insufficient substrates for cellulose synthesis (Haigler et al., 2012; Byrt et al., 2018),This directly limits the thickening and elongation of the secondary cell wall, resulting in thinner fiber cell walls and reduced structural strength (Niu et al., 2015) (Jareczek et al., 2023).Additionally, salt stress disrupts the fiber development process, leading to incomplete or abnormal cellulose deposition, which causes fluctuations in the micronaire value (Ul-Allah et al., 2021; Liu et al., 2025c).

Although DVRT modification improves soil aeration and root expansion capacity (Xia et al., 2026). To some extent, this alleviated physiological drought stress, but it could not fully offset the negative impact of high-concentration brackish water on fiber development. Therefore, future research should focus on finding the “yield-quality” balance point. By optimizing irrigation regimes (such as rotational irrigation and mixed irrigation) combined with Deep vertical rotary tillage techniques, we can utilize non-conventional water resources while minimizing their damage to the microscopic structure of cotton fibers, thereby achieving synergistic improvements in both yield and quality.

It is important to note that while this study established the link between soil water-salt regulation and crop yield, physiological indicators such as root system architecture, root activity, and tissue ion content were not directly measured. The proposed physiological mechanisms are inferred from the observed agronomic traits and supporting literature. Future research should focus on *in-situ* root monitoring and isotopic tracing to quantitatively verify how DVRT alters root physiological plasticity and nutrient uptake kinetics under brackish water irrigation.

## 5 Conclusions

This study, based on field-scale experiments conducted from 2023 to 2024, systematically elucidates the regulatory mechanisms of the coupled system of deep vertical rotary tillage (DVRT) and saline water drip irrigation (SWDI) on soil water-salt conditions and crop productivity in cotton fields of southern Xinjiang. Key findings are as follows: The DVRT-SWDI coupling significantly enhanced soil water storage capacity, with deep vertical rotary tillage combined with medium-mineralized saline water drip irrigation (D3M2) yielding the best results. Deep vertical rotary tillage (D2/D3) effectively broke through the plowpan, promoting salt leaching to deeper layers and significantly reducing salt accumulation in the 0–60 cm root zone, with D3M1 exhibiting the best salt-blocking effect. Conversely, shallow tillage combined with high-salinity water (D1M3) showed negative interactions, increasing the risk of deep soil salinization. Deep plowing combined with low/medium salinity brackish water (M1/M2) significantly promoted cotton growth. Treatments D2M2 and D3M2 achieved substantial yield increases by optimizing the root zone environment, with yields showing a year-on-year upward trend. Concurrently, appropriate deep plowing patterns (D3M1, D2M1) effectively mitigated salt stress on fiber quality, enabling synergistic improvements in both yield and quality. Based on TOPSIS comprehensive evaluation and response surface modeling (RSM) optimization, D3M2 was confirmed as the optimal combination for comprehensive benefits. Multi-objective optimization identified optimal agronomic parameters as: Deep vertical rotary tillage depth of 40–45 cm and saline water drip irrigation salinity of 2.82–3.29 g·L^-1^. This parameter range effectively balances high yield and quality objectives while maintaining sound soil water-salt equilibrium. The findings provide scientific basis and technical support for saline-alkali land remediation and safe utilization of saline water drip irrigation in arid regions. Adaptive adjustments based on local soil and climate conditions are recommended during implementation.

## Data Availability

The original contributions presented in the study are included in the article/**Supplementary Material**. Further inquiries can be directed to the corresponding author/s.
