# Supplementary material for: Coupling effects of deep vertical rotary tillage and brackish water irrigation on soil water-salt dynamics and cotton productivity in arid regions
Source: Front Plant Sci. 2026 Mar 2;17:1787141. doi: 10.3389/fpls.2026.1787141 (PMC12989620; doi:10.3389/fpls.2026.1787141)
Supplement: Supplementary file 1 [file Table1.docx]

Coupling Effects of Deep Vertical Rotary Tillage and Brackish Water Irrigation on Soil Water-Salt Dynamics and Cotton Productivity in Arid Regions

Qiang Meng ^a,b^ , Ning Su ^a,b^, Hongguang Liu ^a,b*^, Pengfei Li ^a,b^, Tangang Wang ^c^, Rui Fang^d^ , Yongfu Wu^d^

^a^College of Water Conservancy & Architectural Engineering, Shihezi University, Shihezi 832000, China

^b^Key Laboratory of Modern Water–Saving Irrigation of Xinjiang Production & Construction Group, Shihezi 832000, China

^c^Institute of Agricultural Science of the Third Division of Xinjiang Production and Construction Corps, Tumxuk 843900, China

^d^ Hydrology and Water Resources Management Center of the Second Division of Xinjiang Production and Construction Corps, Tiemenguan, Xinjiang 841007, China

*Corresponding author.

Email: liuhongguang-521@163.com (H. Liu)

*Supplementary Tables*

Table S1. Physical and chemical properties of the soil in the experimental area.

Table S2. Soil nutrient content in the experimental area.

Table S3. Irrigation and fertilization schedule for the 2023–2024 reproductive period.

Table S4. Quality of irrigation water in the experimental area.

Table S5. Weighted Standardized Matrix (2023-2024).

Table S1. Physical and chemical properties of the soil in the experimental area.

| soil depth  cm | Soil fraction /% | | | Soil texture | Soil bulk  density  g·cm^−3^ | Saturated hydraulic conductivity  mm·min^−1^ | pH value | Soil Salinity  g·kg^−1^ |
| --- | --- | --- | --- | --- | --- | --- | --- | --- |
|  | Sand particles | Silt particles | Clay particles |  |  |  |  |  |
|  | 0.05–2 /mm | 0.05–0.002 /mm | ˂0.002 /mm |  |  |  |  |  |
| 0-20 | 19.00 | 72.13 | 8.87 | Silty loam | 1.45 | 0.70 | 7.9 | 6.55 |
| 20-40 | 14.96 | 75.22 | 9.82 | Silty loam | 1.50 | 0.50 | 8.0 | 5.94 |
| 40-60 | 12.72 | 80.73 | 10.05 | Silty loam | 1.53 | 0.40 | 8.0 | 5.35 |
| 60-80 | 36.15 | 58.21 | 5.64 | Silty loam | 1.60 | 0.45 | 7.9 | 5.22 |
| 80-100 | 51.85 | 42.95 | 5.20 | Silty loam | 1.62 | 0.40 | 7.8 | 5.17 |

*Note*: Soil particles were graded according to the U.S. System of Soil Classification Standards (USDA).

Table S2. Soil nutrient content in the experimental area.

| Soil depth | SOM | ANH | APH | APO |
| --- | --- | --- | --- | --- |
| cm | mg·kg^−1^ | mg·kg^−1^ | mg·kg^−1^ | mg·kg^−1^ |
| 0–20 | 15.54 | 48.32 | 17.85 | 253.12 |
| 20–40 | 13.01 | 38.21 | 12.19 | 246.75 |
| 40–60 | 10.38 | 24.04 | 6.96 | 222.08 |

*Note*: SOM is soil organic matter, ANH is alkaline nitrogen, APH is rapidly available phosphorus, APO is rapidly available potassium.

Table S3. Irrigation and fertilization schedule for the 2023–2024 reproductive period

| Times | Date | DAS | Irr. | Fer. | Date | DAS | Irr. | Fer. |
| --- | --- | --- | --- | --- | --- | --- | --- | --- |
|  |  | (d) | (mm) | (kg·ha^−1^) |  | (d) | (mm) | (kg·ha^−1^) |
|  | 2023 | | | | 2024 | | | |
| 1 | 6.10 | 45 | 37.5 | 40 | 6.12 | 45 | 37.5 | 40 |
| 2 | 6.21 | 56 | 37.5 | 80 | 6.23 | 56 | 37.5 | 80 |
| 3 | 6.28 | 63 | 37.5 | 80 | 6.30 | 63 | 37.5 | 80 |
| 4 | 7.6 | 71 | 57.5 | 130 | 7.8 | 71 | 57.5 | 130 |
| 5 | 7.13 | 78 | 57.5 | 130 | 7.15 | 78 | 57.5 | 130 |
| 6 | 7.20 | 85 | 57.5 | 140 | 7.22 | 85 | 57.5 | 140 |
| 7 | 7.27 | 92 | 57.5 | 140 | 7.29 | 92 | 57.5 | 140 |
| 8 | 8.3 | 99 | 57.5 | 140 | 8.5 | 99 | 57.5 | 140 |
| 9 | 8.10 | 106 | 57.5 | 140 | 8.12 | 106 | 57.5 | 140 |
| 10 | 8.25 | 121 | 22.5 |  | 8.27 | 121 | 22.5 |  |
| Total |  |  | 480 | 1020 |  |  | 480 | 1020 |

*Note:* DAS, days after sowing, Irr is irrigation amount, Fer is fertilization amount.

Table S4. Quality of irrigation water in the experimental area.

| Source | pH | TDS  （g·L^-1^） | Na^+^  （g·L^-1^） | K^+^  （g·L^-1^） | Ca^2+^  （g·L^-1^） | Mg^2+^  （g·L^-1^） | Cl^-^  （g·L^-1^） | SO_4_^2-^  （g·L^-1^） | HCO_3_^-^  （g·L^-1^） |
| --- | --- | --- | --- | --- | --- | --- | --- | --- | --- |
| Irrigation  water | 7.452 | 1.00 | 0.206 | 0.015 | 0.056 | 0.042 | 0.256 | 0.362 | 0.134 |
| 2 g·L^-1^ brackish water | 7.768 | 2.00 | 0.655 | 0.023 | 0.188 | 0.094 | 0.425 | 0.406 | 0.045 |
| 4 g·L^-1^ brackish water | 8.056 | 4.00 | 1.321 | 0.043 | 0.357 | 0.183 | 0.856 | 0.812 | 0.085 |
| 6 g·L^-1^ brackish water | 8.423 | 6.00 | 1.982 | 0.066 | 0.534 | 0.272 | 1.287 | 1.223 | 0.128 |

Table S5. Weighted Standardized Matrix (2023-2024).

| Index Name | D1M1 | D2M1 | D3M1 | D1M2 | D2M2 | D3M2 | D1M3 | D2M3 | D3M3 |
| --- | --- | --- | --- | --- | --- | --- | --- | --- | --- |
| Seedling emergence  rate | 0.0000 | 0.0205 | 0.0356 | 0.0255 | 0.0695 | 0.0598 | 0.0552 | 0.0528 | 0.0524 |
| Average Length  of Aboveground Part | 0.0408 | 0.0572 | 0.0613 | 0.0204 | 0.0408 | 0.0531 | 0.0000 | 0.0327 | 0.0490 |
| Evenness | 0.0520 | 0.0660 | 0.0700 | 0.0140 | 0.0460 | 0.0600 | 0.0000 | 0.0380 | 0.0540 |
| Specific strength  at break | 0.0428 | 0.0571 | 0.0625 | 0.0214 | 0.0410 | 0.0535 | 0.0000 | 0.0286 | 0.0500 |
| Micronaire Value | 0.0000 | 0.0142 | 0.0142 | 0.0709 | 0.0425 | 0.0284 | 0.1276 | 0.0851 | 0.0567 |
| Elongation Rate | 0.0480 | 0.0720 | 0.0480 | 0.0720 | 0.0480 | 0.0720 | 0.0000 | 0.0240 | 0.0240 |
| Cotton dry mass | 0.0251 | 0.0376 | 0.0834 | 0.0581 | 0.1458 | 0.1334 | 0.0067 | 0.0363 | 0.0000 |
| Seed cotton yield | 0.0245 | 0.0367 | 0.0816 | 0.0572 | 0.1428 | 0.1306 | 0.0082 | 0.0355 | 0.0000 |
| WUE | 0.0245 | 0.0367 | 0.0816 | 0.0572 | 0.1428 | 0.1306 | 0.0082 | 0.0355 | 0.0000 |
| SSAR | 0.0601 | 0.0659 | 0.1060 | 0.0315 | 0.0401 | 0.0716 | 0.0000 | 0.0057 | 0.0401 |
| 2023 | | | | | | | | | |
| Seedling emergence  rate | 0.0000 | 0.0109 | 0.0217 | 0.0542 | 0.0651 | 0.0759 | 0.0383 | 0.0492 | 0.0600 |
| Average Length  of Aboveground Part | 0.0372 | 0.0605 | 0.0745 | 0.0233 | 0.0372 | 0.0512 | 0.0000 | 0.0140 | 0.0279 |
| Evenness | 0.0344 | 0.0630 | 0.0763 | 0.0210 | 0.0363 | 0.0534 | 0.0000 | 0.0153 | 0.0305 |
| Specific strength  at break | 0.0301 | 0.0602 | 0.0753 | 0.0226 | 0.0377 | 0.0527 | 0.0000 | 0.0151 | 0.0301 |
| Micronaire Value | 0.0223 | 0.0148 | 0.0000 | 0.0593 | 0.0445 | 0.0223 | 0.0816 | 0.0668 | 0.0445 |
| Elongation Rate | 0.0365 | 0.0731 | 0.1096 | 0.0365 | 0.0731 | 0.0731 | 0.0000 | 0.0000 | 0.0365 |
| Cotton dry mass | 0.0355 | 0.0461 | 0.0851 | 0.0638 | 0.1382 | 0.1276 | 0.0000 | 0.0107 | 0.0036 |
| Seed cotton yield | 0.0355 | 0.0461 | 0.0852 | 0.0639 | 0.1384 | 0.1278 | 0.0000 | 0.0106 | 0.0035 |
| WUE | 0.0355 | 0.0461 | 0.0852 | 0.0639 | 0.1384 | 0.1278 | 0.0000 | 0.0106 | 0.0035 |
| SSAR | 0.0495 | 0.0542 | 0.0918 | 0.0306 | 0.0330 | 0.0589 | 0.0000 | 0.0047 | 0.0330 |
| 2024 | | | | | | | | | |
